# M2 bone marrow-derived macrophage-derived exosomes shuffle microRNA-21 to accelerate immune escape of glioma by modulating PEG3

**DOI:** 10.1186/s12935-020-1163-9

**Published:** 2020-03-27

**Authors:** Fan Yang, Tiecheng Wang, Peng Du, Haitao Fan, Xushuai Dong, Hua Guo

**Affiliations:** 1 Department of Neurosurgery, Shandong Provincial Hospital Affiliated to Shandong University, 324 Jingwuweiqi Road, Jinan, 250021 Shandong China; 2 Department of Neurosurgery, The People’s Hospital of Huantai, Zibo, 256400 Shandong China; 3 Department of Neurosurgery, Shandong Provincial Third Hospital, Jinan, 250021 Shandong China

**Keywords:** Glioma, M2 bone marrow-derived macrophage, Exosomes, MicroRNA-21, Paternally expressed gene 3, Proliferation, Apoptosis

## Abstract

**Background:**

Growing studies have focused on the role of microRNA-21 (miR-21) in glioma, thus our objective was to discuss the effect of M2 bone marrow-derived macrophage (BMDM)-derived exosomes (BMDM-Exos) shuffle miR-21 on biological functions of glioma cells by regulating paternally expressed gene 3 (PEG3).

**Methods:**

Seventy-one cases of human glioma tissues and 30 cases of non-tumor normal brain tissues were collected and stored in liquid nitrogen. PEG3 and miR-21 expression in glioma tissues was tested. The fasting venous blood of glioma patients and healthy control was collected and centrifuged, and then the supernatant was stored at − 80 °C refrigerator. The contents of interferon (IFN)-γ and transforming growth factor-β1 (TGF-β1) in serum were tested by ELISA. Glioma cells and normal glial cells were cultured to screen the target cells for further in vitro experiments. BMDM-Exos was obtained by ultra-high speed centrifugation and then was identified. BMDM-Exos was co-cultured with U87 cells to detect the biological functions. The fasting venous blood of glioma patients was extracted and treated with ethylene diamine tetraacetic acid-K2 anti-freezing, and then CD8^+^T cells were isolated. CD8^+^T cells were co-cultured with U87 cells to detect the CD8^+^T proliferation, cell cytotoxic activity, U87 cell activity, as well as IFN-γ and TGF-β1 levels. Moreover, BALB/c-nu/nu mice was taken, and the human-nude mouse glioma orthotopic transplantation model was established with U87 cells, and then mice were grouped to test the trends in tumor growth. The brain of mice (fixed by 10% formaldehyde) was sliced to detect the expression of Ki67 and proliferating cell nuclear antigen (PCNA). The spleen of mice was taken to prepare single-cell suspension, and the percentage of T lymphocytes in spleen to CD8^+^T cells was detected.

**Results:**

PEG3 expression was decreased and miR-21 expression was increased in glioma cells and tissues. Depleting miR-21 or restoring PEG3 suppressed growth, migration and invasion as well as accelerated apoptosis of glioma cells, also raised CD8^+^T proliferation, cell cytotoxic activity, and IFN-γ level as well as decreased U87 cell activity and TGF-β1 level. BMDM-Exos shuttle miR-21 promoted migration, proliferation and invasion as well as suppressed apoptosis of glioma cells by reducing PEG3. Exosomes enhanced the volume of tumor, Ki67 and PCNA expression, reduced the percentage of CD8^+^T cells in glioma mice.

**Conclusion:**

BMDM-Exos shuffle miR-21 to facilitate invasion, proliferation and migration as well as inhibit apoptosis of glioma cells via inhibiting PEG3, furthermore, promoting immune escape of glioma cells.

## Background

Glioma is the commonest and deadly tumor of the central nervous system, can be divided into grades I to IV in line with the degree of malignancy in compliance with the World Health Organization (WHO) classification [1]. The glioma clinical management faces many challenges despite significant advances in its combination therapy, including surgery, radiotherapy and chemotherapy [2]. The prognostic factors for glioma consist of age, Karnofsky performance status (KPS) score, histologic grade, tumor necrosis, preoperative duration of symptoms, surgical resection extent and multimodal therapeutic strategies [3]. Glioma is characterized by diffusion and high invasiveness, but its potential mechanisms is still far from understanding [4]. Therefore, it is an urgent need to find new and effective strategies and establish potential therapeutic targets for the treatment of human glioma.

Macrophage is a critical component of the immune system, diffusely distributed in the body, expressing various bioactive substances, receptors and enzymes [5]. M2 macrophage ordinarily triggers cell proliferation, extracellular matrix synthesis and tissue remodeling [6]. A study has reported that M2 phenotype macrophages secret transforming growth factor-β1 to promote the migration and stemness of glioma cells [7]. Exosomes are a species of endogenous extracellular vesicles with a diameter of 30–100 nm [8]. A study has reported that exosomes accelerate simultaneous therapy and imaging of glioma in vivo and in vitro [9]. Another study has revealed a potential therapeutic role of exosomes with microRNA-21 (miR-21)-sponge construct in a rat model of glioblastoma [10]. It has been demonstrated that non-coding RNAs act as drivers of the phenotypic plasticity of esophageal mucosa [11]. A study has revealed that long non-coding RNAs are involved in gastroesophageal cancers [12]. miRs are a series of endogenous non-coding RNAs with tumor-inhibiting or tumor-promoting functions [13]. It has been demonstrated that the absolute plasma levels of miR-483-5p in patients with adrenocortical cancer are strong molecular markers [14]. A study has revealed that plasmatic miR-21 is directly linked to myocardial fibrosis and related to left ventricular functional and structural impairment [15]. Another study has reported that miR-21 expression is up-regulated in pancreatic adenocarcinoma [16]. It is displayed that miR-21 facilitates proliferation and suppresses senescence and apoptosis of glioma cells [17]. A study has also presented that miR-21 expression is linked to overall survival of glioma patients [18]. Paternally expressed gene 3 (PEG3) is the founding member of the 500-kb imprinting domain located on the proximal mouse chromosome 7/human chromosome 19q13.4 [19]. A study has suggested that hyper-methylation of the PEG3 promoter in primary human gliomas results in loss of imprinting and reduced PEG3 mRNA expression, which is related to tumor grade [20]. It has been demonstrated that epigenetic silencing and loss of imprinting of PEG3 is implicated in tumorigenesis of glioma [21]. In this study, we therefore examined the role of M2 bone marrow-derived macrophage (BMDM)-derived exosomes (BMDM-Exos) shuffle miR-21 on glioma immune escape by regulating PEG3.

## Materials and methods

### Compliance with ethical standards

The study was approved by the Institutional Review Board of Shandong Provincial Hospital Affiliated to Shandong University. All participants signed a document of informed consent. All animal experiments were tally with the Guide for the Care and Use of Laboratory Animal by International Committees.

### General information

From May, 2011 to May, 2014, a total of 71 fresh glioma specimens with complete clinical data from Shandong Provincial Hospital Affiliated to Shandong University were collected, and the fresh peripheral blood samples of the same patient were collected at the same time. All the patients were diagnosed with primary glioma without any relevant treatment before surgical resection. Patients with severe infections and other types of tumors were excluded. The clinical data were gathered through the archived cases of pathology department, all the patients were followed up, and the survival time was recorded. The peripheral blood of 30 healthy examiners matched with age and gender ratio of glioma patients were collected. In addition, 30 cases of normal brain tissues (resected from decompression surgery in patients with acute traumatic brain injury) were collected as the control group. Glioma was graded histologically on the basis of the WHO classification criteria of central nervous system. All the tissue samples were immediately preserved in liquid nitrogen. The expression of miR-21 and PEG3 in tissues were tested by reverse transcription quantitative polymerase chain reaction (RT-qPCR) and western blot assay, and the correlation with clinicopathological features of glioma patients was analyzed.

### CD8^+^T cell sorting, activation and amplification

The fasting venous blood of glioma patients was extracted and treated with ethylene diamine tetraacetic acid (EDTA)-K2 anti-freezing. Mini MACS immunomagnetic separation system was adopted, CD8^+^T cells were obtained in strictly accordance with the instructions of human CD8^+^T cells magnetic bead separation kit (Miltenyi Biotec Inc., Auburn, CA, USA). Cell purity was analyzed by a flow cytometer. Cells were added into Roswell Park Memorial Institute (RPMI) 1640 medium, and cultured at 37 °C with 5% CO_2_. Cells (1 × 10^6^) were appended with 25 μL Human T-Activator CD3/CD28 and 20 U/mL interleukin (IL)-2 and cultured at 37 °C with 5% CO_2_.

### Acquisition, induction and identification of BMDM

The peripheral blood from healthy volunteers was taken, then diluted with 0.9% normal saline with a ratio of 1: 2 and added with lymphocyte separation medium. The buffy coat was obtained by density gradient centrifugation, and then rinsed 3 times with phosphate buffered saline (PBS). Cells (2 × 10^6^) were seeded into a sterile 24-well plate with serum-free Dulbecco’s Modified Eagle Medium (DMEM) and cultured for 24 h. The un-adherent cells were removed to obtain purified monocytes. After 3 day of culture, the cells stimulated by IL-4 and IL-13 (20 ng/mL) were polarized into M2 BMDM and cultured for 2 day. The cell morphology was observed under an inverted microscope. M2 BMDM related marker protein Arg-1 and CD163 were identified by western blot assay.

After identification, M2 BMDM was detached by trypsin, seeded and cultured in a 6-well plate with 3 × 10^6^ cells/well. When reached 60% confluence, cells were incubated with serum-free medium for 1 h. According to Lipofectamine 2000 transfection reagent (Invitrogen Inc., Carlsbad, CA, USA), miR-21 inhibitors and inhibitors negative control (NC) were co-transfected into M2 BMDM, then corresponding exosomes were extracted. The oligonucleotide sequences were available from GenePharma Ltd. Company (Shanghai, China).

### BMDM-Exos extraction and identification

The collected cell culture supernatant were centrifuged for 10 min at 500×*g* to remove cell precipitation, then centrifuged for 10 min at 2000×*g* to remove cell debris, and filtered with 0.22 μm filter membrane to collect the supernatant, then centrifuged at the ultra-centrifuge tube for 4 h (100,000×*g*) and suspended with PBS. At last, cells were centrifuged for 70 min (100,000×*g*), the obtained precipitation was exosomes.

The exosomes suspension (50 μL) were placed on the 26 cm × 19 cm × 2 cm dissecting pan. A copper mesh with supporting membrane was contacted with the exosomes suspension and placed for 3–5 min. The copper mesh was taken out and air-dried. Three percent phosphotungstic acid solution(50 μL) were dripped on the wax plate, the copper mesh was placed on the surface of 3% phosphotungstic acid staining solution for 3–5 min, then the copper mesh was dried under the filament lamp and pictured by an electron microscope. BMDM-Exos marker protein CD63, CD81 and TSG101 were identified by western blot assay.

### Uptake of exosomes

The glioma U87 cells (3 × 10^4^ cells/well) were seeded on a 24-well pre-loaded with cell slides. After cells adhered to the wall, BMDM-Exos (80 μg/mL) pre-stained with PKH67 (Sigma-Aldrich Chemical Company, St Louis, MO, USA) were cultured in the well plate for 1 day. Cell slides were fixed in 4% paraformaldehyde (PFA) for 20 min, cleaned 3 times with PBS, stained with 4’-6-diamidino-2-phenylindole for 1 h, blocked with anti-fluorescent quenching agent and finally, photographed under the fluorescence microscope.

### Cell culture, grouping and transfection

The cell frozen suspension of glioma cells U87, U251, SHG44, A172, LN299 (the Cell Bank of Type Culture Collection of Chinese Academy of Sciences, Shanghai, China) and normal glial cells HA1800 (Beijing ChuangLian Biology Co., Ltd., Beijing, China) were centrifuged (151×*g*, 5 min) to discard the supernatant. Cells were suspended evenly with DMEM containing 10% fetal bovine serum and cultured with 5 mL complete medium in a 25 cm^2^ culture flask. The medium was changed each 24 h. Cells were detached with 0.25% trypsin and passaged when reached 60–70% confluence, and the logarithmic growth phase cells were selected for the experiment. The expression of miR-21 and PEG3 in each cell line were tested by RT-qPCR and western blot assay. The cells with the largest miR-21 expression difference with HA1800 cells were screened for subsequent experiment.

U87 cell line was divided into 6 groups: inhibitors NC group (transfected with miR-21 inhibitors NC), miR-21 inhibitors group (transfected with miR-21 inhibitors), overexpression (OE)-NC group (transfected with OE-PEG3 NC vector), OE-PEG3 group (transfected with OE-PEG3 vector), inhibitors NC + small interfering RNA (si)-NC group (transfected with miR-21 inhibitors NC and PEG3 siRNA NC) and miR-21 inhibitors + si-PEG3 group (transfected with miR-21 inhibitors and PEG3 siRNA). miR-21 inhibitors and inhibitors NC, OE-PEG3 and OE-NC, PEG3 siRNA and si-NC were purchased from GenePharma.

Glioma cells and BMDM-Exos were co-cultured. Then U87 cells were divided into 4 groups: blank group (not co-cultured with exosomes), Exo group (co-cultured with BMDM-Exos without transfection of any sequence for 48 h), Exo-inhibitors NC group (co-cultured with BMDM-Exos transfected with miR-21 inhibitors NC for 48 h) and Exo-miR-21 inhibitors group (co-cultured with BMDM-Exos transfected with miR-21 inhibitors for 48 h).

Glioma cells were co-cultured with CD8^+^T cells. CD8^+^T cells were grouped into control group (CD8^+^T cells co-cultured with normal U87 cells), inhibitors NC group (CD8^+^T cells co-cultured with U87 cells that had been transfected with miR-21 inhibitors NC), miR-21 inhibitors group (CD8^+^T cells co-cultured with U87 cells that had been transfected with miR-21 inhibitors), OE-NC group (CD8^+^T cells co-cultured with U87 cells that had been transfected with OE-PEG3 vector NC) and OE-PEG3 group (CD8^+^T cells co-cultured with U87 cells that had been transfected with OE-PEG3 vector). Construction of co-culture system: the transfected U87 cells were suspended with RPMI 1640 medium and counted. CD8^+^T cells were diluted with RPMI 1640 medium, the concentration of cell suspension was adjusted to 1000 cells/mL. Cell suspension were spread on a 96-well cell plate with 100 μL per well, and incubated in a 37 °C, 5% CO_2_ incubator for 72 h.

### Enzyme-linked immunosorbent assay (ELISA)

The peripheral blood samples (5 mL) of glioma patients were collected, and 30 healthy examiners were taken as a control. After numbered, the blood was treated with 1.5–2.0 mg/mL EDTA, centrifuged at 151×*g* for 15 min, then the supernatant was preserved and stored at a − 80 °C refrigerator. The supernatant of co-culture CD8^+^T cells was collected, and the concentration of transforming growth factor-β1 (TGF-β1) and interferon (IFN)-γ in serum and cell supernatant were detected by TGF-β1 and IFN-γ kit, respectively (R&D Systems, Minneapolis, MN, USA).

### Carboxyfluorescein diacetate succinimidyl ester (CFSE) labeling assay

CD8^+^T cells in the 96-well plate were extracted into a centrifuge tube and centrifuged with an appropriate amount of PBS, the supernatant was removed, then the cells were added with RPMI 1640 medium. The concentration of cell suspension was set to 1 × 10^7^ cells/mL. Cell suspension was incubated with CFSE solution at a 37 °C, 5% CO_2_ incubator for 20 min, mixed with calf serum, then put at 4 °C for 10 min to stop the staining. The residual CFSE solution was washed away by PBS solution, and cells were diluted to 1 × 10^6^ cells/mL with RPMI 1640 complete medium. The proliferation of CD8^+^T cells were determined by a flow cytometer.

### CD8^+^T cells cytotoxicity test and cell counting kit (CCK)-8 assay

CD8^+^T cells were re-suspended in RPMI 1640 medium containing 10% fast calcification solution, and cell cytotoxic activity was analyzed. U87 cells were used as the target cells and CD8^+^T cells as the effector cells, cell cytotoxic activity was detected at the E: T ratio of 10: 1, 5: 1 and 2.5: 1, separately. CD8^+^T cells and U87 cells were co-cultured in 96-well plates at a specified ratio of lymphocytes to target cells and incubated at 37 °C, 5% CO_2_ for 4 h. The operations were performed in accordance with the instructions for the lactic dehydrogenase (LDH) cytotoxicity test kit (Shanghai Best Biotechnology Co., Ltd., Shanghai, China). Cytotoxicity = (optical density (OD) value of treated sample − OD value of control sample)/(OD value of cell maximum enzyme activity − OD value of control sample).

CD8^+^T cells were extracted from the 96-well plate co-culture system, the residual CD8^+^T cells and metabolites of co-culture were washed off with PBS solution, and cells were added with RPMI 1640 complete medium. CCK-8 solution (20 μL) was added avoiding light, then 100 μL sterile PBS solution was added to the well around the plate, and incubated for 4 h. The OD value was tested in a microplate reader at 570 nm, the cell activity was calculated.

### 5-ethynyl-2′-deoxyuridine (EdU) assay

Cell medium was diluted with EdU solution at 1000: 1 ratio (Guangzhou RiboBio Co., Ltd., Guangdong, China) to prepare 50 μmol/L EdU medium. Each well was incubated with 100 μL EdU medium for 2 h. Cells were washed twice with PBS (5 min per time). Each well was added with 50 μL fixation fluid (PBS containing 4% PFA) and incubated for 30 min, then incubated 5 min with 50 μL 2 mg/mL glycine and 10 min with 100 μL penetrant (PBS containing 0.5% Triton X-100), then incubated 30 min with 100 μL 1 × Apollo staining reaction solution (Beyotime Biotechnology Co., Shanghai, China). Cells were cleaned three times with 100 μL penetrant (10 min per time) and twice with 100 μL methanol (5 min per time). Hoechst 33342 staining reaction solution was diluted by deionized water with a proportion of 100: 1. Each well was incubated with 100 μL 1 × Hoechst 33342 solution for 30 min, then the cells were observed and analyzed under the fluorescence microscope.

### Annexin V-fluorescein isothiocyanate (FITC)/propidium iodide (PI) double staining

Cells in each group were dripped into the 4 mL centrifuge tube, respectively, added with appropriate amount of PBS and centrifuged for 5 min at 151×*g*. The PBS without calcium and magnesium ions was pre-cooled at 4 °C. Cells were suspended and centrifuged for 5 min at 151×*g*. The above-mentioned glioma cells were added with 300 μL 1 × Binding buffer, incubated with 5 μL Annexin V-FITC for 5 min and with 5 μL PI for 5 min. Then cells were tested on the flow cytometer. FloJo software was utilized for data analysis and cartography. Annexin V-FITC/PI kit was purchased from Best Biotechnology.

### Transwell assay

Matrigel (Sigma) was melted at 4 °C and mixed with serum-free medium. Matrigel (60 μL) was diluted and polymerized in the upper chamber of Transwell (Corning Glass Works, Corning, N.Y., USA) for 30 min. The treated U87 cells were detached and centrifuged, then suspended in serum-free DMEM. The lower chamber of Transwell was added with 600 μL DMEM containing 10% bovine serum albumin, and cell suspension (3 × 10^4^ cells) was cultured in the upper chamber containing polymerized Matrigel for 24 h. Then the upper chamber was taken out, fixed 5 min with 4% PFA and stained 5 min with crystal violet staining solution. The cells penetrating through the membrane were counted.

### Scratch test

U87 cells were seeded in a 6-well plate (1 × 10^6^ cells/well). When the cells covered the bottom of the plate, a cross-shaped mark was scratched vertical to the bottom of the plate with a 1 mL pipette tip. The plate was fully rinsed with 37 °C PBS solution and added with the serum-free medium. Five visual fields were randomly selected, and the scratch at 0 h was recorded under the inverted phase contrast microscope. Cells were continuously cultured for 2 day, and the scratches were recorded. Finally, the scratches in 0 h and 48 h were compared, and the cell migration distance was calculated.

### Orthotopic transplantation tumor model of nude mice

Twenty BALB/c-nu/nu nude mice aged 6–8 weeks and weighed 18–20 g were bought from Nanjing Biomedical Research Institute (Nanjing, China). The mice were fed in the animal room with the specific pathogen-free grade environment and treated with adaptive feeding for 7 day. U87 cells were grouped (n = 5) into PBS group (mice injected with PBS solution), +Exo group (mice were injected with BMDM-Exos), +CD8 group (mice were injected with CD8^+^T cells) and +Exo-CD8 group (mice were injected with CD8^+^T cells pretreated by BMDM-Exos). Cells in the logarithmic growth phase were detached by 0.25% trypsin and the digestive fluid was collected, centrifuged, then the supernatant was removed. Cells were washed twice with Hank’s solution and then made into cell suspension. Cells (2 × 10^5^) were seeded in the right caudate nucleus of nude mice. At the 3, 6, 9, 12, 15, 18 day after tumor implantation, mice in the +Exo group were intravenously injected with BMDM-Exos. At the 5 day after tumor implantation, mice in the +CD8 group and the +Exo-CD8 group were injected with CD8^+^T cells or CD8^+^T cells pretreated by BMDM-Exos (twice a week). After 7, 14, 21 and 28 day of tumor implantation, each group of nude mice were examined by magnetic resonance imaging (MRI) scanning to detect the growth of tumor.

The tumor-bearing mice was euthanized at the 28th day, the whole brain was fixed with 10% formaldehyde for 24 h. Immunohistochemical staining was performed with coronal sections (2–3 μm). The primary antibody Ki67 (1:200), proliferating cell nuclear antigen (PCNA) (1:200) were purchased from Abcam Inc. (Cambridge, MA, USA), while secondary antibody Envision was bought from Dako (Glostrup, Denmark). The sections were developed by diaminobenzidine, counterstained by hematoxylin, differentiated by lithium carbonate, dehydrated by gradient ethanol, cleared by xylene, sealed by neutral gum and observed under a light microscope.

The spleens of mice were taken and made into single cell suspension, the red blood cells were lysed by ACK lysate (Lonza, Sins, Switzerland). Then the cells were stained with CD4, CD8α and DX5 antibodies (BD Biosciences, Franklin Lakes, NJ, USA), and incubated at 4 °C for 45 min. Cells were washed with pre-iced PBS and re-suspended. The percentage of T lymphocytes in spleen to CD8^+^T cells was tested in a flow cytometer.

### RT-qPCR

The glioma tissues were lysed on the ice with Trizol. The total RNA in tissues was abstracted in accordance with the steps of “separation-RNA precipitation- RNA washing-RNA re-dissolution”. The concentration of RNA was determined. The same amount of total RNA was reversely transcripted into complementary DNA (cDNA). Similarly, the cells of each group were placed on ice, the supernatant was discarded, and the cells were lysed by adding Trizol lysate. The reverse transcription way was the same as above. The cDNA was utilized for RT-qPCR. The primers were composed by BGI Co., Ltd. (Shenzhen, Guangdong, China) (Table 1). The relative expression of miR-21 and PEG2 was standardized by U6 or β-actin. PCR 9700 was adopted for analysis. The data was analyzed by 2^−ΔΔCt^ method.

**Table 1:** Primer sequence

Gene	Sequence (5′→3′)
miR-21	Forward: 5′-CCGGCCTAGCTTATCAGACTG-3′
	Reverse: 5′-AGTGCAGGGTCCGAGGTA-3′
PEG3	Forward: 5′-CTGAGGTTGGAACGGACATC-3′
	Reverse: 5′-CTAGCTGAAGGTTGGAACGG-3′
U6	Forward: 5′-CTCGCTTCGGCAGCACA-3′
	Reverse: 5′-AACGCTTCACGAATTTGCGT-3′
β-actin	Forward: 5′-AAGTGTGACGTGGACATCCG-3′
	Reverse: 5′-CCTTCCAGCAGATGTGGATC-3′

*miR-21* microRNA-21, *PEG3* paternally expressed gene 3

### Western blot assay

Protein in glioma tissues and cells were collected. The protein concentration was determined by bicinchoninic acid protein quantitative kit. The protein was boiled with 6 × loading buffer for 5 min at 95 °C. The sample was prepared and stored at − 20 °C. According to the molecular weight of the target protein, the corresponding concentration of sodium dodecyl sulfate polyacrylamide gel electrophoresis was prepared, 50 μg protein was used for electrophoresis separation, and then the protein was transferred to the nitrocellulose membrane. The membrane was blocked for 2 h, and incubated with primary antibodies Arg-1 (1:2000, Abcam, Cambridge, UK), CD163 (1:2000, Abcam), PEG3 (1:1000, Abcam), TSG101 (1:1000), CD63 (1:1000) CD81 (1:500, Santa Cruz Biotechnology, Inc, Santa Cruz, CA, USA), Bcl-2 (1:1000), Bax (1:1000), matrix metalloproteinase (MMP)-2 (1:1000), MMP-9 (1:1000, Cell Signaling Technologies, Beverly, MA, USA) and β-actin (1:3000, ZSGB-Bio, Beijing, China) overnight, then incubated with secondary antibody (1:5000, ZSGB-Bio) labeled by horseradish peroxidase for 1 h. Enhanced chemiluminescence was used for development.

### Dual luciferase reporter gene assay

The website (http://www.microrna.org/microrna/) was used to predict the target relationship between PEG3 and miR-21. Dual luciferase reporter gene assay was utilized for verify that PEG3 was the direct target gene of miR-21. PEG3 3′untranslated region (UTR) gene fragment was composed, and introduced into luciferase reporter gene plasmid psiCHECK-2 (Promega, Madison, Wisconsin, USA) with endonuclease sites XhoI and NotI, and wild type (PEG3-WT) recombinant dual luciferase reporter plasmid was constructed. A complementary sequence mutation site of a seed sequence was devised on the basis of PEG3-WT to compose a PEG3 mutant type (MUT). The MUT fragment was inserted into the psiCHECK-2 plasmid by endonuclease cleavage of restriction enzyme with T4 DNA ligase, and MUT (PEG3-MUT) recombinant dual luciferase reporter plasmid was constructed. The correctly sequenced luciferase reporter plasmid WT, MUT and mimics NC, and miR-21 mimics were co-transfected into U87 cells for reporter gene analysis. Fifty μL luciferase analysis buffer II was placed in a specific glass tube, then 20 μL cell lysate was added in, the average luminous value F1 was read in the luminescence measurements, then 50 μL Stop&Glo reagent was placed in luminescence measurements to read the average luminous value F2. The regulation effect of miR-21 to PEG3 was judged according to F1/F2 ratio.

### Statistical analysis

All data were analyzed by SPSS 21.0 software and GraphPad Prism 5. The measurement data distributed normally were represented by mean ± standard deviation. The independent *t* test was performed for comparisons between two groups, one-way analysis of variance (ANOVA) was used for comparisons among multiple groups and Tukey’s multiple comparisons test was used for pairwise comparisons after one-way ANOVA. Survival analysis was verified by Kaplan–Meier test, and the correlation of miR-21 and PEG3 mRNA with clinicopathological features of glioma patients was identified by Chi square test. *p* value < 0.05 was indicative of statistically significant difference.

## Results

### PEG3 expression is decreased and miR-21 expression is raised in glioma cells and tissues

**Fig. 1:**
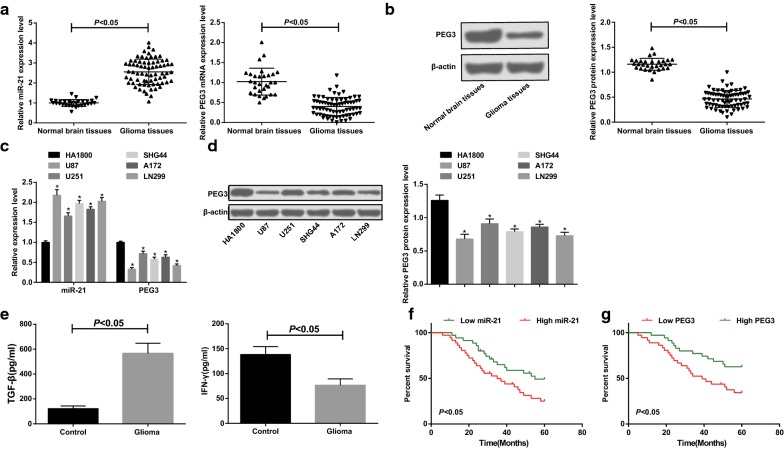
PEG3 expression is decreased and miR-21 expression is elevated in glioma cells and tissues. **a** Comparison of miR-21 and PEG3 mRNA expression between glioma tissue (n = 71) and normal brain tissue (n = 30). **b** Comparison of protein expression of PEG3 in glioma tissues (n = 71) and normal brain tissues (n = 30). **c** Comparison of miR-21 and PEG3 mRNA expression in HA1800, U87, U251, SHG44, A172 and LN299 cells (N = 3). **d** Comparison of PEG3 protein expression in HA1800, U87, U251, SHG44, A172 and LN299 cells (N = 3). E, Comparison of TGF-β1 and IFN-γ levels in peripheral blood of patients with glioma (n = 71) and healthy subjects (n = 30). F, Survival analysis curve of the effect of miR-21 on prognosis of glioma patients. G, Survival analysis curve of the effect of PEG3 mRNA on prognosis of patients with glioma. *p < 0.05 vs. the HA1800 cells. The measurement data were represented by mean ± standard deviation. The independent t-test was performed for comparisons between two groups, one-way ANOVA was used for comparisons among multiple groups and Tukey’s multiple comparisons test was used for pairwise comparisons after one-way ANOVA. Kaplan–Meier and log-rank test was used for survival analysis

Western blot assay and RT-qPCR were utilized to test miR-21 and PEG3 expression in glioma tissues and cells as well as normal brain tissues and normal glioma cells. The results in tissues demonstrated that (Fig. 1a, b) in contrast with the normal brain tissues, miR-21 expression was elevated and PEG3 expression was reduced in glioma tissues (both *p *< 0.05). It was displayed that (Fig. 1c, d) in relation to HA1800 cells, miR-21 expression was up-regulated and PEG3 expression was down-regulated in U87, U251, SHG44, A172 and LN299 cells (all *p *< 0.05).

ELISA results reported that (Fig. 1e) in relation to the peripheral blood of healthy examiners, TGF-β1 content was increased and IFN-γ content was decreased in peripheral blood of glioma patients (both *p *< 0.05).

### miR-21 and PEG3 expression are linked to WHO classification and tumor size of glioma patients

The glioma patients were divided into low expression group and high expression group according to the median level of relative expression of miR-21 and PEG3. The effect of miR-21 and PEG3 expression on survival and prognosis of glioma patients were analyzed by Kaplan–Meier, and it was reported that (Fig. 1f, g) the prognosis was poor in glioma patients with high expression of miR-21 and low expression of PEG3 (*p *< 0.05).

The results of the correlation between the expression of miR-21/PEG3 mRNA and the clinicopathological features of glioma patients showed that (Table 2): the proportions of up-regulated miR-21 and down-regulated PEG3 were increased when the WHO classification was in III-IV stage and the tumor size was more than 5 cm in glioma patients, which meant that the expression of miR-21 and PEG3 were closely associated with WHO classification and tumor size of glioma patients (both *p *< 0.05), but not to age, gender, tumor location and KPS score of glioma patients (all *p *> 0.05).

**Table 2:** Relationship between relative expression of miR-21 and PEG3 and clinicopathological features in patients with glioma

Clinicopathological data	n	miR-21 expression		*p*	PEG3 expression		*p*
		Low expression group (n = 35)	High expression group (n = 36)		Low expression group (n = 36)	High expression group (n = 35)	
Age (year)				0.188			0.736
< 50	44	19	25		23	21	
≥ 50	27	16	11		13	14	
Gender				0.559			0.288
Male	39	18	21		22	17	
Female	32	17	15		14	18	
Tumor location				0.196			0.168
Forehead	22	13	9		10	12	
Crown	19	6	13		12	7	
Pulvinar	16	7	9		10	6	
Temple	14	9	5		4	10	
WHO classification				0.005			0.001
I + II	41	26	15		14	27	
III + IV	30	9	21		22	8	
KPS score				0.281			0.120
< 70	33	14	19		20	13	
≥ 70	38	21	17		16	22	
Tumor size (cm)				0.024			0.006
< 5	37	23	14		13	24	
≥ 5	34	12	22		23	11	

The data in this table were enumeration data, which were tested by Chi square test

*miR-21* microRNA-21, *PEG3* paternally expressed gene 3, *WHO* World Health Organization, *KPS* Karnofsky performance status

### Depleting miR-21 suppresses growth, migration and invasion as well as accelerates apoptosis of glioma cells

**Fig. 2:**
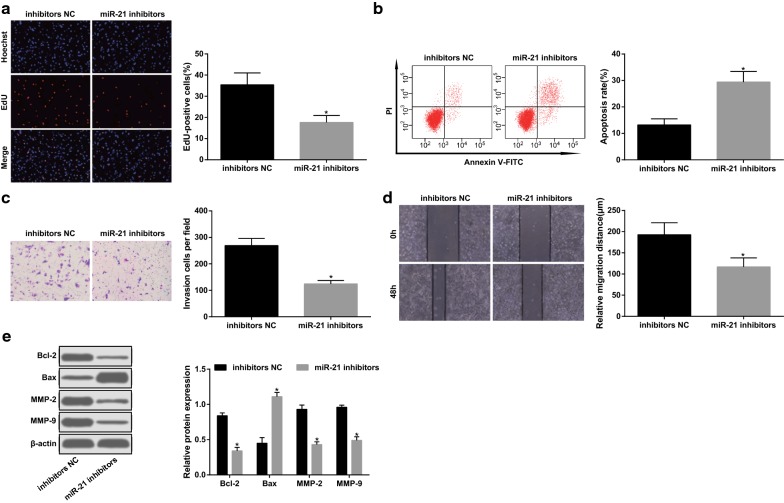
Depleting miR-21 suppresses growth, migration and invasion as well as accelerates apoptosis of glioma cells. **a** Cell proliferation tested by EdU assay. **b** Detection of apoptosis by flow cytometry. **c** Cell invasion tested by Transwell assay. **d** Cell migration detected by scratch test. **e** The protein expression of Bcl-2, Bax, MMP-2 and MMP-9 measured by western blot assay. N = 3. *p < 0.05 vs. the inhibitors NC group. The measurement data were represented by mean ± standard deviation. Comparison between two groups were assessed by t test

EdU assay, flow cytometry, Transwell assay, scratch test and western blot assay were adopted to detect the proliferation, invasion, migration, apoptosis, and Bcl-2, Bax, MMP-2 and MMP-9 protein expression in glioma cells, the results presented that versus the inhibitors NC group, the proliferative, invasive and migratory capacities as well as the protein expression of Bcl-2, MMP-2 and MMP-9 were reduced, while apoptosis rate and Bax protein expression were enhanced in the miR-21 inhibitors group in U87 cells (all *p* < 0.05) (Fig. 2a–e).

### PEG3 is targeted by miR-21 and restoring PEG3 represses migration, proliferation and invasion as well as advances apoptosis of glioma cells

**Fig. 3:**
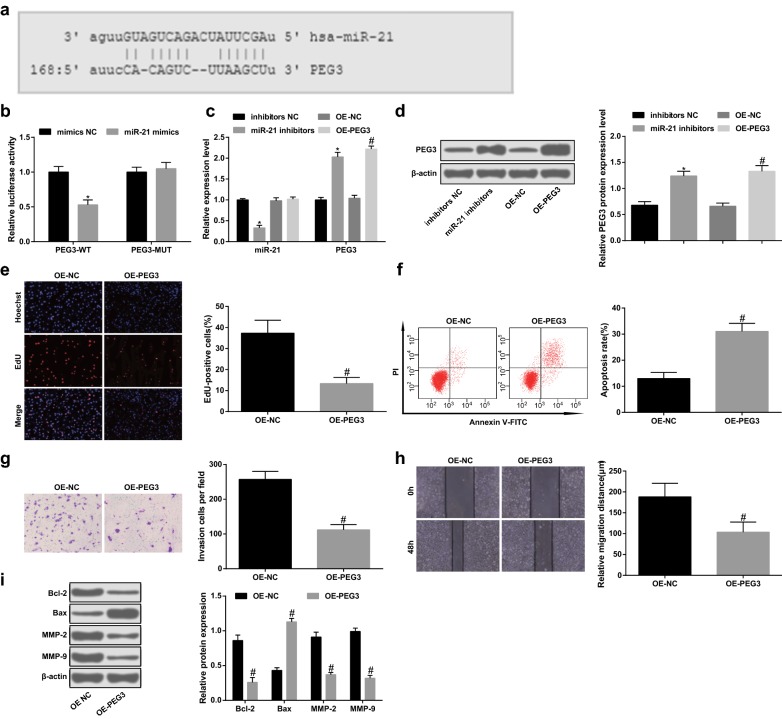
PEG3 is targeted by miR-21 and restoring PEG3 represses migration, proliferation and invasion as well as advances apoptosis of glioma cells. A, The binding site of miR-21 and PEG3 predicted by microRNA.org bioinformatics software. B, The target relationship between miR-21 and PEG3 confirmed by dual luciferase reporter gene assay. C, The expression of miR-21 and PEG3 mRNA in cells detected by RT-qPCR. D, Detection of PEG3 protein expression in cells by western blot assay. E, Cell proliferation tested by EdU assay. F, Detection of apoptosis by flow cytometry. G, Cell invasion tested by Transwell assay. H, Cell migration detected by scratch test. I, The protein expression of Bcl-2, Bax, MMP-2 and MMP-9 measured by western blot assay. N = 3. *p < 0.05 vs. the inhibitors NC group. ^#^*p* < 0.05 vs. the OE-NC group. The measurement data were represented by mean ± standard deviation. Comparison between two groups were assessed by t test

Bioinformatics software MicroRNA.org was used to predict the binding site of miR-21 and PEG3 (Fig. 3a), it was revealed that there existed a binding site of miR-21 in PEG3 3′UTR. Dual luciferase reporter gene assay further showed that (Fig. 3b) after co-transfection of PEG3-WT with miR-21 mimics into U87 cells, the luciferase activity of cells was dramatically reduced (*p* < 0.05), and after co-transfection of PEG3-MUT with miR-21 mimics, the luciferase activity of cells showed no marked difference (*p* > 0.05).

In vitro cell test revealed that compared to the inhibitors NC group, miR-21 expression was decreased and PEG3 expression was heightened in the miR-21 inhibitors group (both *p* < 0.05). In relation to the OE-NC group, miR-21 expression didn’t apparently change (*p *> 0.05) but PEG3 expression was elevated in the OE-PEG3 group (*p* < 0.05, Fig. 3c, d). By comparison with the OE-NC group, the proliferation, invasion and migratory capacities as well as Bcl-2, MMP-2 and MMP-9 protein expression were decreased while apoptosis rate and Bax protein expression were increased in the OE-PEG3 group (all *p* < 0.05) (Fig. 3e–i).

### Down-regulated PEG3 effectively reverses the effect of poor expression of miR-21 on glioma cells

**Fig. 4:**
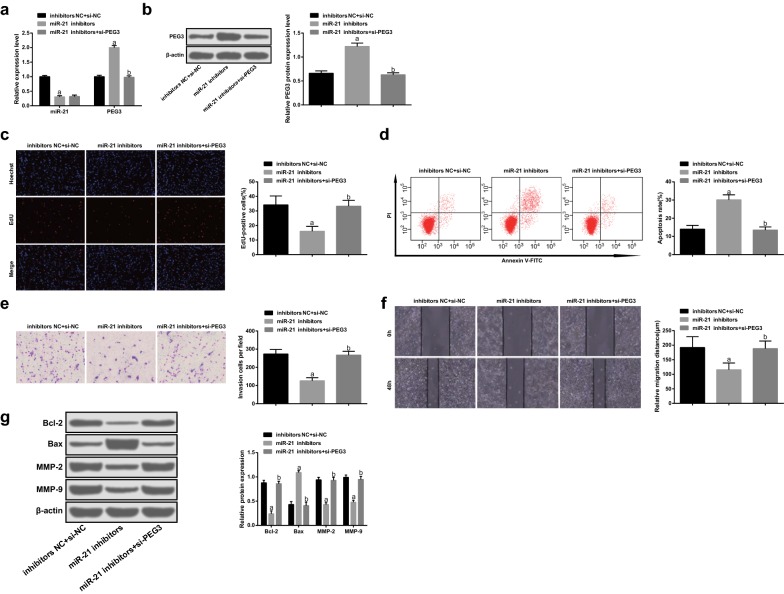
Down-regulated PEG3 reverses the effect of poor expression of miR-21 on glioma cells. **a** The expression of miR-21 and PEG3 mRNA in cells detected by RT-qPCR. **b** Detection of PEG3 protein expression in cells by western blot assay. **c** Cell proliferation tested by EdU assay. **d** Detection of apoptosis by flow cytometry. **e** Cell invasion tested by Transwell assay. **f** Cell migration detected by scratch test. **g** The protein expression of Bcl-2, Bax, MMP-2 and MMP-9 measured by western blot assay. N = 3. a *p* < 0.05 vs. the inhibitors NC + si-NC group. b *p* < 0.05 vs. the miR-21 inhibitors group. The measurement data were represented by mean ± standard deviation. Comparison among multiple groups were conducted by one-way ANOVA followed by Tukey’s multiple comparisons test

In vitro cell experiments displayed that versus the inhibitors NC + si-NC group, miR-21 expression was decreased and PEG3 expression was increased in the miR-21 inhibitors group (both *p* < 0.05). In comparison to the miR-21 inhibitors group, miR-21 expression didn’t broadly vary (*p* > 0.05) and PEG3 expression was decreased in the miR-21 inhibitors + si-PEG3 group (*p* < 0.05) (Fig. 4a, b).

The proliferation, invasion and migratory capacities as well as Bcl-2, MMP-2 and MMP-9 protein expression were decreased while apoptosis rate and Bax protein expression were increased in the miR-21 inhibitors group relative to that in the inhibitors NC + si-NC group (all *p* < 0.05). In relation to the miR-21 inhibitors group, the proliferation, invasion and migratory capacities as well as Bcl-2, MMP-2 and MMP-9 protein expression were heightened as well as apoptosis rate and Bax protein expression were decelerated in the miR-21 inhibitors + si-PEG3 group (all *p* < 0.05) (Fig. 4c–g).

### Identification of M2 BMDM and its exosomes

**Fig. 5:**
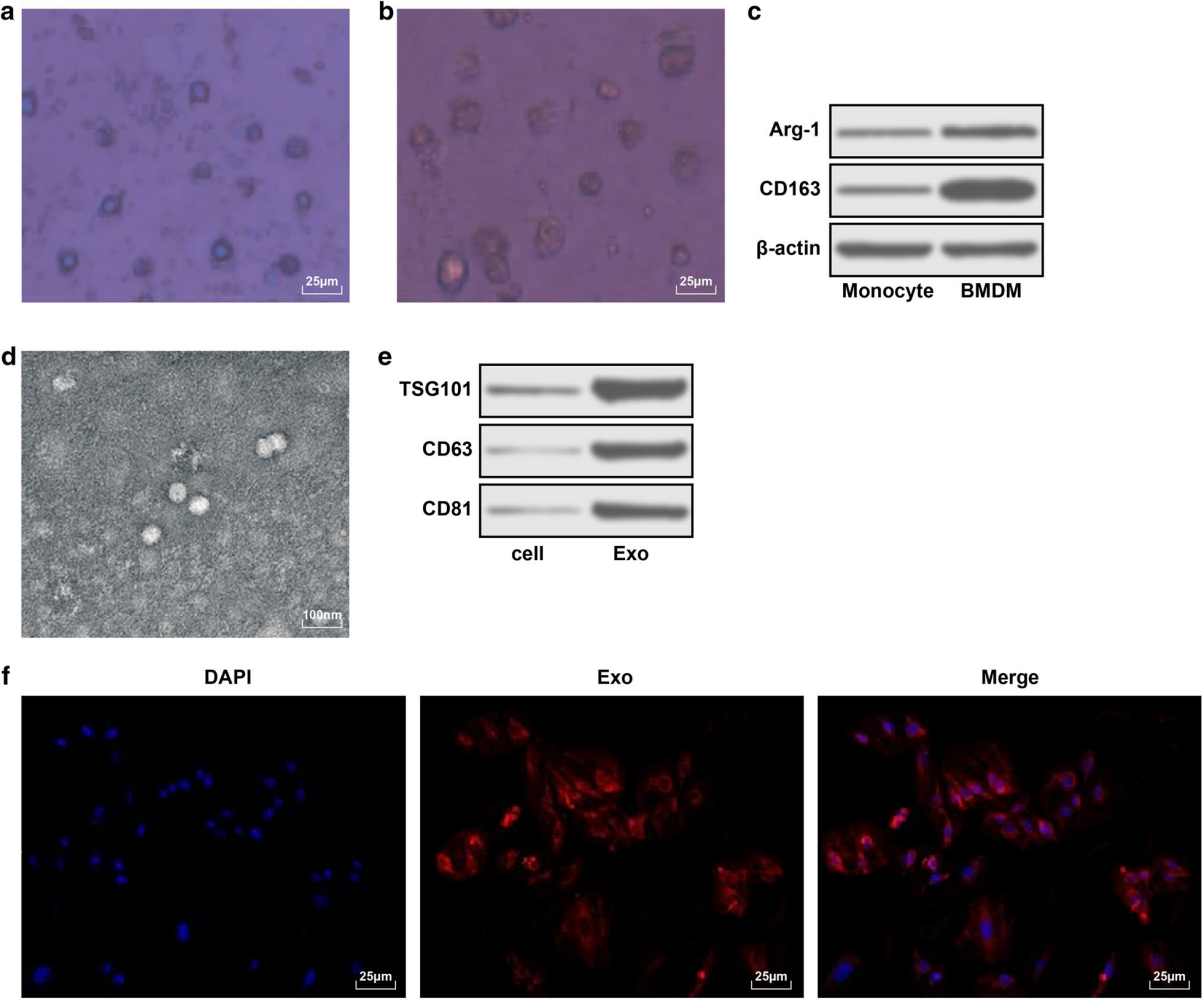
Identification of M2 BMDM and its exosomes. **a** Morphology of macrophages without IL-4 and IL-13 polarization (scale bar: 25 μm). **b** Morphology of macrophages stimulated by IL-4 and IL-13 polarization (scale bar: 25 μm). **c** Expression of Arg-1 and CD163 proteins in polarized and un-polarized-stimulated macrophages. **d** BMDM-Exos morphology under transmission electron microscope (scale bar: 100 nm). **e** Expression of exosomes marker proteins TSG101, CD63 and CD81. F, Exosomes uptake test (scale bar: 25 μm)

As shown in Fig. 5a, b, after 2 day of IL-4 and IL-13 polarization, the volume of macrophages was larger than that of un-polarized macrophages, the wrinkle on the surface of cells was increased and the heteromorphism was obvious. Western blot assay results presented that (Fig. 5c) the expression of Arg-1 and CD163 (markers of M2 BMDM) in macrophages polarized by IL-4 and IL-13 were remarkably raised.

Under the transmission electron microscope, it could be seen that the exosomes had uniform size and regular shape (circular or elliptical vesicles) with a diameter of 50–100 nm (Fig. 5d), and had distinct exosomes marker proteins TSG101, CD63 and CD81 (Fig. 5e). Under the fluorescence microscope, the marked exosomes entered the U87 cells, after 24 h of culture, the exosomes had entered the U87 cells and mostly gathered in the nuclear membrane (Fig. 5f).

### BMDM-Exos shuttled miR-21 promotes proliferation, migration and invasion as well as inhibits apoptosis of glioma cells by reducing PEG3

**Fig. 6:**
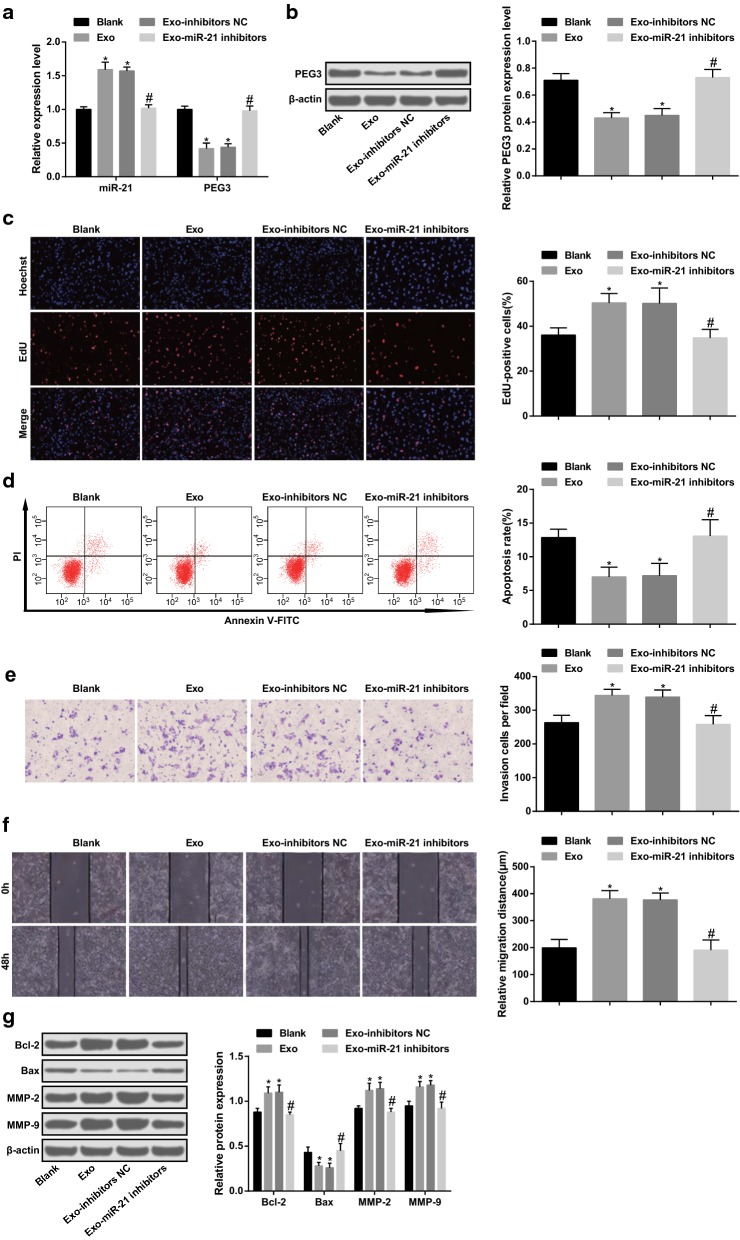
BMDM-Exos shuttle miR-21 promotes proliferation, migration and invasion as well as inhibits apoptosis of glioma cells by reducing PEG3. **a** miR-21 and PEG3 mRNA expression in cells detected by RT-qPCR. **b** PEG3 protein expression in cells detected by western blot assay. **c** Cell proliferation tested by EdU assay. **d** Detection of apoptosis by flow cytometry. **e** Cell invasion tested by Transwell assay. **f** Cell migration detected by scratch test. **g** The protein expression of Bcl-2, Bax, MMP-2 and MMP-9 measured by western blot assay. n = 3. *p < 0.05 vs. the blank group. ^#^*p* < 0.05 vs. the Exo-inhibitors NC group. The measurement data were represented by mean ± standard deviation. Comparison among multiple groups were conducted by one-way ANOVA followed by Tukey’s multiple comparisons test

After co-cultured with BMDM-Exos, versus the blank group, miR-21 expression was elevated and PEG3 expression was reduced glioma cells in the Exo group (both *p* < 0.05). In relation to the Exo-inhibitors NC group, miR-21 expression was decreased and PEG3 expression was increased in the Exo-miR-21 inhibitors group (both *p* < 0.05). No evident difference could be observed in miR-21 and PEG3 expression between the Exo group and Exo-inhibitors NC group (both *p* > 0.05). In contrast with the blank group, the proliferation, invasion and migration capacities as well as Bcl-2, MMP-2 and MMP-9 protein expression were enhanced as well as apoptosis rate and Bax protein expression were reduced in the Exo group (all *p* < 0.05). The proliferation, invasion and migration capacities as well as Bcl-2, MMP-2 and MMP-9 protein expression were decreased as well as apoptosis rate and Bax protein expression were raised in the Exo-miR-21 inhibitors group relative to that in the Exo-inhibitors NC group (all *p* < 0.05) (Fig. 6a–g).

### Depleted miR-21 and restored PEG3 raise CD8^+^T proliferation, cell cytotoxic activity, and IFN-γ level as well as decline U87 cell activity and TGF-β1 level

**Fig. 7:**
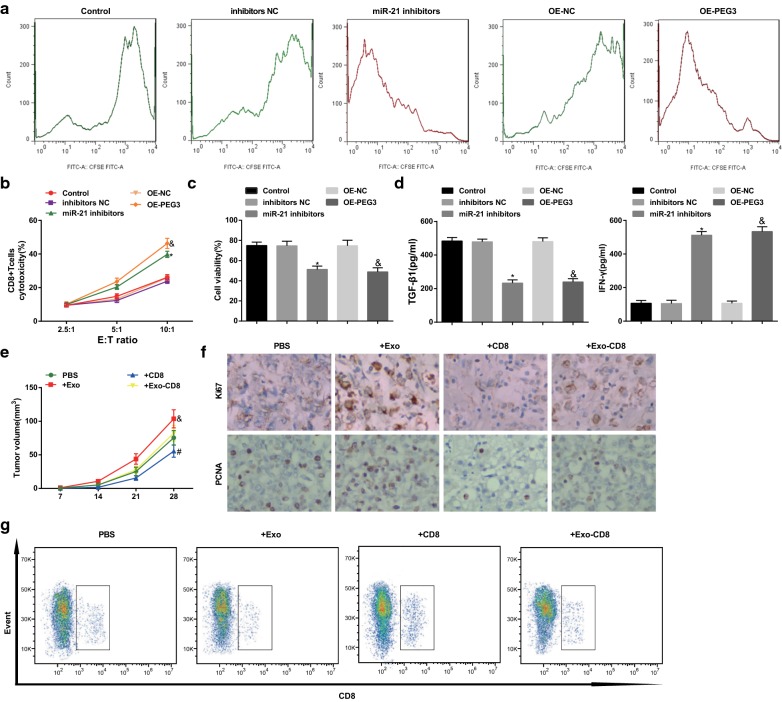
Depleted miR-21 and restored PEG3 raise CD8^+^ T proliferation, cell cytotoxic activity, and IFN-γ level as well as decline U87 cell activity and TGF-β1 level; exosomes enhances the volume of tumor, Ki67 and PCNA expression as well as reduces the percentage of CD8^+^ T cells in glioma mice. **a** The proliferation of CD8^+^T cells tested by CFSE. **b** Detection of glioma cytotoxic activity by LDH. **c** Cell activity of U87 cells tested by CCK-8 assay. **d** TGF-β1 and IFN-γ levels tested by ELISA. **e** Tumor growth curve of mice in each group. **f** Immunohistochemical staining of Ki76 and PCNA in mouse orthotopic transplantation tumor (25 μm). **g** Percentage of T lymphocytes in mouse spleen to CD8^+^T cells detected by flow cytometry. *p < 0.05 vs. the inhibitors NC group. ^$^*p* < 0.05 vs. the OE-NC group. ^&^*p* < 0.05 vs. the PBS group. ^#^*p* < 0.05 vs. the +CD8 group. The measurement data were represented by mean ± standard deviation. Comparison among multiple groups were conducted by one-way ANOVA followed by Tukey’s multiple comparisons test

CFSE, LDH cytotoxicity test, CCK-8 assay and ELISA were adopted to test CD8^+^T cells proliferation, cell cytotoxic activity, U87 cell activity, as well as TGF-β1 and IFN-γ levels. It was presented that (Fig. 7a–d) contrasted with the inhibitors NC group and the OE-NC group, CD8 ^+^ T cell proliferation, cell cytotoxic activity, and IFN-γ level were promoted while U87 cell activity and TGF-β1 level were decreased in the miR-21 inhibitors group and the OE-PEG3 group (all *p* < 0.05).

### Exosomes enhances the volume of tumor and expression levels of Ki67 and PCNA as well as reduces the percentage of CD8^+^T cells in glioma mice

MRI scanning was utilized for testing the volume of tumor, the results displayed that (Fig. 7e) in contrasted with the PBS group and the +CD8 group, the volume of tumor was enhanced in the +Exo group and the +Exo-CD8 group (all *p* < 0.05). Immunohistochemical staining results reported that (Fig. 7f) versus the PBS group and the +CD8 group, Ki67 and PCNA expression levels were elevated in the +Exo group and the +Exo-CD8 group (all *p* < 0.05). Flow cytometry was adopted to test the percentage of T lymphocytes in spleen to CD8^+^T cells, it was demonstrated that (Fig. 7g) in comparison to the PBS group and the +CD8 group, the percentage of CD8^+^T cells was reduced in the +Exo group and the +Exo-CD8 group (all *p* < 0.05).

## Discussion

Glioma is the most common and invasive primary tumor in the nervous system, accounting for about 80% of primary malignant brain tumors [22]. It is customarily considered that miR-32 targeting PTEN raises M2 macrophage polarization in the glioma microenvironment as well as further accelerates the development of glioma [23]. A previous study has reported that ultrasound promotes naturally equipped exosomes derived from blood serum and macrophages for orthotopic glioma treatment [24]. A recent study has provided a proof that the expression of miR-21 is modulated by β-catenin/STAT3 pathway and facilitates glioma cell invasion [25]. Also, it was demonstrated that PEG3 is one of the candidate tumor inhibitor genes for glioma [26]. As the related mechanisms of exosomal miR-21 in glioma remains to be explored, the objective of our study was to investigate the role of BMDM-Exos shuffle miR-21 on biological functions of glioma by regulating PEG3.

The body’s immune system is composed of immune organs, immune cells and immune molecules. Immune organs are divided into central and peripheral immune organs, among which the central immune organs mainly include bone marrow and thymus, while the peripheral immune organs mainly include spleen, lymph nodes and mucosa-related lymphoid tissues. Lymphocytes play a very important role in the body’s immune system, among which a considerable proportion of T lymphocytes can be divided into CD4^+^ and CD8^+^ subsets mainly according to their functional characteristics and surface marks. CD4^+^T cells mainly differentiate into three types of effector cells, namely Th1, Th2 and Th17 cells, they secrete different cytokines to play different immune effects. CD8^+^T cells are cytotoxic T cells, which are also the effector cells that play a key role in anti-tumor effect [27]. The spleen is the most important place for T lymphocytes to temporarily store the peripheral immune organs. The size and physiological function of the spleen can affect the number and immune function of T lymphocytes to a certain extent [28]. Thus, we use mice/s spleen to test the percentage of the T lymphocytes.

In this study, this study suggested high expression of miR-21 and low expression of PEG3 in glioma cells and tissues. Consistent with our study, a study has reported that the expression of miR-21 was significantly raised in blood of patients affected by glioma [29]. Another study has presented that miR-21 expression was dramatically elevated in malignant glioma cells relative to that in the normal human glial cells [30]. A previous study has revealed that the expression of PEG3 was remarkably decreased in glioma cell lines [21]. It has been suggested that the expression of PEG3 was obviously reduced in glioma cell lines [26]. Collectively, the expression of miR-21 and PEG3 is in conformity with the previous study findings to some extent. Moreover, it was demonstrated that PEG3 is targeted by miR-21. According to Li et al., miR-93-3p targeted PEG3 in cervical cancer [31]. It was also presented that PEG3 is directly regulated by miR-514a-3p [32]. However, the target relationship between PEG3 and miR-21 in glioma has not been uncovered before. Moreover, our study has revealed that miR-21 and PEG3 expression were linked to WHO classification and tumor size of glioma patients. Due to limitation of known researches, this correlation needs further study.

In addition, it was revealed in this paper that depleting miR-21 or restoring PEG3 suppressed growth, migration and invasion as well as boosted apoptosis of glioma cells. In addition, this study also demonstrated that depleting miR-21 or restoring PEG3 raised CD8^+^T cell proliferation, cell cytotoxic activity, and IFN-γ level as well as decreased U87 cell activity and TGF-β1 level. It has been suggested previously that down-regulation of miR-21 restrained proliferation of glioma cells and tumor growth in vivo [33]. Another study has verified that poor expression of miR-21 reduced proliferation but facilitated apoptosis of glioma cells [22]. It was reported that miR-21 inhibitors in glioma cells decreased cell proliferation, migration and invasion as well as promoted the apoptosis [34]. Other study also displayed that depleted miR-21 attenuated glioma cell proliferation and accelerated cell apoptosis [35]. It was revealed that depleted miR-21 meaningfully decreased cell viability of kidney cancer cells [36]. A study reported that miR-21 down-regulation inactivated TGF-β1 in liver fibrosis [37]. Moreover, it was indicated in our study that restoring PEG3 had the same effect of depleting miR-21 on glioma. It is revealed that up-regulation of PEG3 protein reduced proliferation and advanced apoptosis of human glioma stem cells [20]. A study also reported that PEG3 elevation suppressed cell cycle entry and invasion in vitro as well as restrained tumor growth and metastasis in vivo in cervical cancer [31]. Furthermore, our study also showed that exosomes promoted proliferation, migration and invasion as well as inhibited apoptosis of glioma cells, enhanced the volume of tumor and Ki67 and PCNA expression as well as reduced the percentage of CD8^+^T cells in glioma mice. It was reported that glioma cells exosomes deliver miR-148a to accelerate proliferation and metastasis of glioblastoma [38]. Other study also reported that glioma associated-human mesenchymal stem cells-derived exosomes facilitate glioma stem cells proliferation and clonogenicity in vitro as well as tumorigenicity in vivo [39]. As presented in the above findings, it is easily noticed that miR-21 inhibition and PEG3 restoration are both play a suppressive role in diseases. Moreover, our study revealed that BMDM-Exos shuffling miR-21 could promote immune escape of glioma cells. It is demonstrated that glioma-stem cells secreted exosomes can function as mediators of intercellular communication to facilitate tumor immune escape [40]. It has been suggested that miR-21 is critical for the immune response [41]. A study has reported that miRs in tumor exosomes promote immune escape in melanoma [42]. However, the relation of BMDM-Exos shuffling miR-21 with immune escape of glioma cells has not been discussed, which need further exploration. Furthermore, in our study, in vivo experiments has verified the results of in vitro experiments.

## Conclusion

In conclusion, our study provides evidence that BMDM-Exos shuffle miR-21 to facilitate invasion, proliferation and migration as well as inhibit apoptosis of glioma cells via inhibiting PEG3, furthermore, promoting immune escape of glioma cells. This study suggests that miR-21/PEG3 axis could be a network that is helpful for the diagnosis and treatment of glioma. In addition, investigation of BMDM-Exos carrying miR-21 yields a better understanding for glioma treatment. However, the research is still at the preclinical stage. Thus, it is also recommended that similar experiments are needed in order to further explore the intrinsic mechanisms.

## Data Availability

Not applicable.

## Abbreviations

miR-21: MicroRNA-21
BMDM: Bone marrow-derived macrophage
PEG3: Paternally expressed gene 3
TGF-β1: Transforming growth factor-β1
PCNA: Proliferating cell nuclear antigen
WHO: World Health Organization
RT-qPCR: Reverse transcription quantitative polymerase chain reaction
RPMI: Roswell Park Memorial Institute
DMEM: Dulbecco’s Modified Eagle Medium
NC: Negative control
PFA: Paraformaldehyde
ELISA: Enzyme-linked immunosorbent assay
IFN: Interferon
CFSE: Carboxyfluorescein diacetate succinimidyl ester
CCK: Cell counting kit
LDH: Lactic dehydrogenase
FITC: Annexin V-fluorescein isothiocyanate
PI: Propidium iodide
SPF: Pecific pathogen-free
MRI: Magnetic resonance imaging
PCNA: Proliferating cell nuclear antigen
UTR: 3′ untranslated region
MUT: Mutant type
ANOVA: Analysis of variance
